# The Medical Society

**Published:** 1899-10-28

**Authors:** 


### THE MEDICAL SOCIETY.

The meeting of the Medical Society last Monday was devoted to the consideration of the infectivity of malignant growths, the subject being introduced by a paper by Dr. Washbourn and Mr. Bellingham Smith. A considerable amount of evidence was brought forward to show that malignant growths may not only be infective within the person or the animal attacked, but that, in exceptional circumstances, the disease is capable of transference to other animals. Although the cause of such growths cannot be said to have, as yet, been cleared up, there seems much reason for believing that the origin of the mischief is a parasite. There was much in the discussion to suggest that malignancy is due to an infection by morbid cells containing within them the parasite on which their morbid condition depends. What seemed to be brought out pretty clearly was that the mere infection of the organism with a parasite would not account for the clinical phenomena of cancer. No mere local reaction to an irritant would, for example, set up an epithelial growth in parts far removed from epithelium. It is the epithelium itself which invades the organism, and the question is, what is the cause of this new and morbid activity which enables epithelial cells to invade the lymphatic spaces and be carried to other parts? The suggestion is that this perverted action is the result of the development of certain parasites, possibly of the nature of blastomycetes, within the cells; and, further, that this may be a general cause of malignancy, setting up cancer when acting upon epithelium, and sarcoma, when such growth takes place in the cells of connective tissue structures. This aspect of the matter widens the field very considerably. At the same time it would seem to simplify the conception.

